# Tumor Molecular Features Predict Endometrial Cancer Patients’ Survival After Open or Minimally Invasive Surgeries

**DOI:** 10.3389/fonc.2021.634857

**Published:** 2021-02-26

**Authors:** Yibo Dai, Jingyuan Wang, Luyang Zhao, Zhiqi Wang, Jianliu Wang

**Affiliations:** Department of Obstetrics and Gynecology, Peking University People’s Hospital, Beijing, China

**Keywords:** endometrial neoplasms, minimally invasive surgical procedures, molecular features, survival, recurrence

## Abstract

**Background:**

The Cancer Genome Atlas (TCGA) project shed light on the vital role of tumor molecular features in predicting endometrial cancer patients’ prognosis. This study aims to investigate the survival impact of surgical approaches on patients with different genetic alterations.

**Methods:**

473 endometrial cancer patients from TCGA database were selected. To analyze the prognostic impact of surgical approach, survival analyses were conducted in patients with different molecular features. Finally, a simplified molecular stratification model was established to select patients suitable for open or minimally invasive surgery (MIS).

**Results:**

In our cohort, 291 patients received open surgery and 182 received MIS. Molecular features influenced patients’ survival after different surgical approaches. Based on survival analyses, three molecular subtypes were generated, with subtype 1 harboring *POLE* mutation (*POLE^mt^*), microsatellite-instability high (MSI-H), homologous recombination repair (HRR) pathway mutation or *MUC16* mutation (*MUC16^mt^*); subtype 3 carrying *TP53* mutation; and subtype 2 without specific molecular feature. The survival influence of molecular subtypes depended on surgical approaches. In the open surgery cohort, three subtypes showed similar survival outcome, while in the MIS cohort, prognosis varied significantly among three subtypes, with subtype 1 the best and subtype 3 the worst. In stepwise Cox regression, molecular subtype was an independent predictor of recurrence-free survival in patients receiving MIS (*p* < 0.001).

**Conclusion:**

The molecular features of endometrial cancer are associated with patients’ prognosis after different surgical approaches. MIS should be recommended in patients with *POLE^mt^*, MSI-H, HRR pathway mutation or *MUC16^mt^*, while for patients with *TP53* mutation, open surgery is better concerning oncological safety.

## Introduction

Endometrial cancer is one of the most common gynecologic malignancies both in western countries and around the world (1, 2). During the past decades, some vital clinical trials, including LAP2 (3, 4), GOG 258 (5), the Post-Operative Radiation Therapy in Endometrial Carcinoma (PORTEC) serial trials (6–8), etc., have been carried out for refining endometrial cancer treatment, with a goal of designing individualized surgical and adjuvant therapy strategy. In particular, the advantages of minimally invasive surgery (MIS) over open surgery have been proved in two large trials, LAP2 and the Laparoscopic Approach to Cancer of the Endometrium (LACE) trial (3, 4, 9, 10). In a meta-analysis including 9 studies, the non-inferiority of total laparoscopic hysterectomy (TLH) versus total abdominal hysterectomy (TAH) regarding endometrial cancer patients’ long term survival was further demonstrated (11).

Until now, the treatment of endometrial cancer is basically determined by clinicopathological staging and patients’ risk stratification. But in recent years, increasingly more attention has been paid to tumor molecular features. In 2013, the Cancer Genome Atlas (TCGA) research network proposed four molecular subtypes of endometrial cancer based on multi-omics analysis (12), which linked molecular features to endometrial cancer patients’ survival outcomes. Besides, data about molecular targeted therapies and immunotherapies in patients with certain genetic alterations are accumulating. And in the currently ongoing PORTEC-4a trial, researchers are trying to decide radiotherapy strategy based on patients’ molecular risk profile in stage I endometrial cancer (13). These lead us to reconsider the possible survival influence of surgical approaches (open surgery vs. MIS) on patients with distinct molecular features.

Previously, our study proved that tumor microsatellite status influenced the recurrence of endometrial cancer after different surgical approaches (14). This retrospective study based on TCGA data aims to further group patients by certain genetic alterations and analyze their survival outcome after MIS or open surgeries.

## Methods

### Data Sources, Genetic Information, and Patient Selection

In the study, the clinical and genetic data of TCGA Uterine Corpus Endometrial Carcinoma (UCEC) project were downloaded from the Genome Data Commons (GDC) Data Portal (https://portal.gdc.cancer.gov), the UCSC-Xena platform (15) and cBioPortal (16, 17). According to previous studies (12, 18–21), six vital genetic features related to patients’ prognosis (*POLE*, microsatellite status, homologous recombination repair [HRR] pathways, *MUC16, CTNNB1, TP53*) were selected for study. HRR mutation was defined as missense mutations, nonsense mutations, insertions, deletions or splice mutations in genes of HRR related pathways, as reported by the previous study (22) (see **Table S1** for the entire gene list used for deciding HRR mutation in the study).

There were totally 548 patients in the database. No patient had a history of neoadjuvant therapy. Five patients with colorectal cancer history, 24 without information of surgical approach and 46 with incomplete genetic information were excluded, and finally 473 eligible patients were selected for further analysis. The study protocol was exempted by the Institutional Review Board of Peking University People’s Hospital since only deidentified data from a public database was used.

### Term Definitions

The definitions of all clinicopathological terms were based on those defined in the Common Data Element (CDE) Browser 5.3.5 (https://cdebrowser.nci.nih.gov). The American Joint Committee on Cancer (AJCC) Clinical Group Stage system was adopted for the staging of all patients, and the stage of each case was decided according to the version used when the diagnosis was made. For neoplasm grade, there were four different values in the original dataset (G1, G2, G3 and high grade). In accordance with the FIGO criteria (23), G1, G2, and G3 were used to describe well-differentiated, moderately-differentiated and poorly-differentiated tumors, respectively. Since high grade was used to describe tumor samples that exhibit poorly differentiated or undifferentiated cells, cases with a value of high grade were reclassified as G3 in the analysis. Besides, deep myometrial invasion was defined as invasion depth of the tumor ≥50% of the whole thickness of the myometrium.

### Follow-Up and Endpoint Measures

The median follow-up time for eligible patients was 30.6 months (interquartile range: 18.0 - 56.1 months). We used overall survival (OS) and recurrence-free survival (RFS) as two measures of patients’ survival outcomes. For the cases in TCGA database, OS and RFS were determined from when initial pathological diagnosis of endometrial cancer was made to when death or disease recurrence occurred, respectively. In all survival analyses, cases without any endpoint events were censored at their last follow-up.

### Statistical Analysis

In the study, χ^2^ test and Fisher’s exact test were performed for comparing categorical variables, and student *t* test was used for comparing continuous variables. The Venn diagram of the distribution of genetic features among eligible patients was drawn using the jvenn online tool (24). Kendall correlation analysis was used to see the correlation between different features. To analyze the survival outcome of patients receiving different surgical approaches, Kaplan-Meier survival analyses (log rank test) were conducted. The results of Kaplan-Meier method were further verified by Cox proportional hazard models. Propensity score matching (PSM) was used for adjusting baseline characteristics. In PSM, a clipper width of 0.02 and a match ratio of 1:1 were adopted. Multivariate stepwise Cox regression (method: backward: conditional; entry criteria: *p* < 0.05; removal criteria: *p* > 0.10) was used to reveal independent survival risk factors for the open surgery cohort and the MIS cohort. In stepwise regression, variables with *p* < 0.05 in univariate analyses were included (lymph node metastasis was excluded for its overlap with disease stage, and postoperative adjuvant therapy was also excluded for collinearity with multiple clinicopathological factors and significant influence on the models). In all Cox regression models, the proportional hazard hypothesis was tested with time-dependent covariates. Statistical analyses were performed using Statistics Package for the Social Sciences (SPSS) software (version 22.0; IBM Corporation, Armonk, NY, USA) and R software version 3.5.3 (https://www.r-project.org/). In all analyses, two-sided *p* values were used, and *p* values less than 0.05 were considered statistically significant.

## Results

### Surgical Information and Molecular Characteristics

Among all the eligible patients, 291 (61.5%) received open surgery and 182 (38.5%) received MIS. Two surgical approaches did not show significant differences in lymph node resection rate and extent (**Table S2**). The distribution of six genetic features (*POLE* mutation [*POLE^mt^*], microsatellite-instability high [MSI-H], HRR mutation, *MUC16* mutation [*MUC16^mt^*], *CTNNB1* mutation [*CTNNB1^mt^*] and *TP53* mutation [*TP53^mt^*]) were comparable between the two groups (**Table 1**). Besides, the distributions of *POLE^mt^*, MSI-H, HRR mutation, *MUC16^mt^* showed significant overlap, and were highly correlated with each other in the entire cohort (**Figure S1**).

**Table 1:** Genetic features of 473 patients by surgical approach^a^.

Characteristic	Surgical approach		*P* Value^a^
	Open surgery (N = 291)	MIS (N = 182)	
***POLE^mt^*, No.(%)**			0.285
No	242 (83.2)	158 (86.8)	
Yes	49 (16.8)	24 (13.2)	
**MSI-H, No.(%)**			0.847
No	199 (68.4)	126 (69.2)	
Yes	92 (31.6)	56 (30.8)	
**HRR mutation, No.(%)**			0.755
No	146 (50.2)	94 (51.6)	
Yes	145 (49.8)	88 (48.4)	
***MUC16^mt^*, No.(%)**			0.728
No	210 (72.2)	134 (73.6)	
Yes	81 (27.8)	48 (26.4)	
***CTNNB1^mt^*, No.(%)**			0.425
No	211 (72.5)	138 (75.8)	
Yes	80 (27.5)	44 (24.2)	
***TP53^mt^*, No.(%)**			0.286
No	181 (62.2)	122 (67.0)	
Yes	110 (37.8)	60 (33.0)	

MIS, minimally invasive surgery; POLE^mt^, POLE mutation; MSI-H, microsatellite-instability high; HRR, homologous recombination repair; MUC16^mt^, MUC16 mutation; CTNNB1^mt^, CTNNB1 mutation; TP53^mt^, TP53 mutation. ^a^ χ^2^ test.

### Survival Influence of Surgical Approaches in Patients With Different Molecular Features

**Figure 1:**
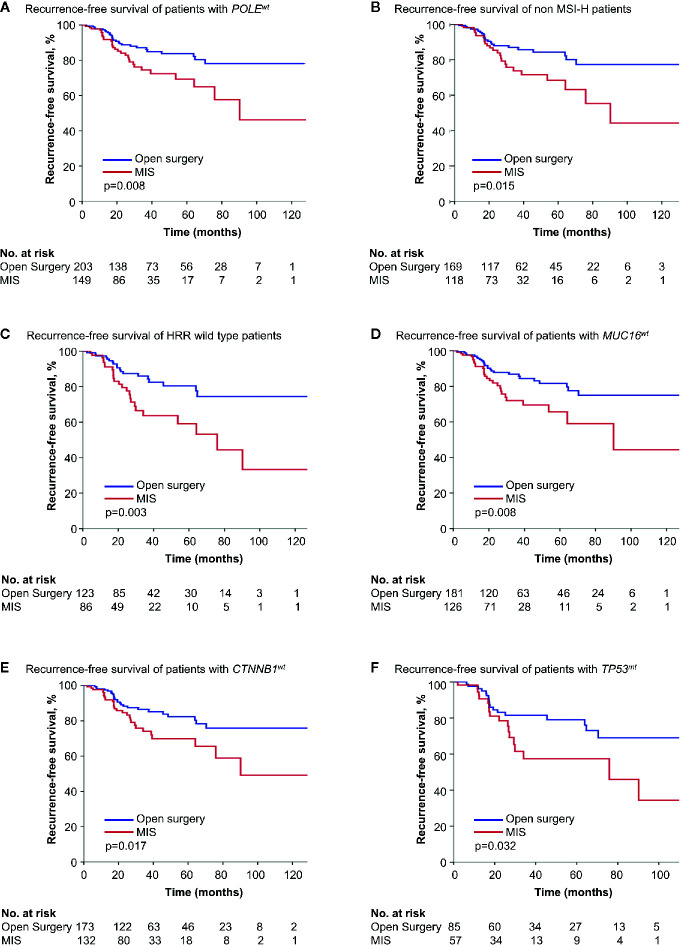
Kaplan-Meier survival curves of patients with different genetic features by surgical approach. **(A)** Recurrence-free survival of patients with *POLE^wt^*. **(B)** Recurrence-free survival of non MSI-H patients. **(C)** Recurrence-free survival of HRR with wild type patients. **(D)** Recurrence-free survival of patients with *MUC16^wt^*. **(E)** Recurrence-free survival of patients with *CTNBB1^wt^*. **(F)** Recurrence-free survival of patients with *TP53^wt^*. MIS, minimally invasive surgery; *POLE^wt^*, *POLE* wild type; MSI-H, microsatellite-instability high; HRR, homologous recombination repair; *MUC16^wt^*, *MUC16* wild type; *CTNNB1^wt^*, *CTNNB1* wild type; *TP53^mt^*, *TP53* mutation.

We divided endometrial cancer cases according to the status of the six genetic features, respectively, and the survival influence of surgical approach in each subgroup was analyzed. In Kaplan-Meier survival analyses, minimally invasive surgery was associated with shorter RFS in *POLE* wild type (*POLE^wt^*), non MSI-H, HRR wild type, *MUC16* wild type (*MUC16^wt^*), *CTNNB1* wild type (*CTNNB1^wt^*), or *TP53^mt^* patients (*p* = 0.008, 0.015, 0.003, 0.008, 0.017 and 0.032) (**Figure 1**). But in the counterpart cohorts, the survival outcome after two surgeries was similar. Cox regressions validated the results above (**Table S3**).

### Survival Influence of Surgical Approaches in Four TCGA Molecular Subtypes

**Figure 2:**
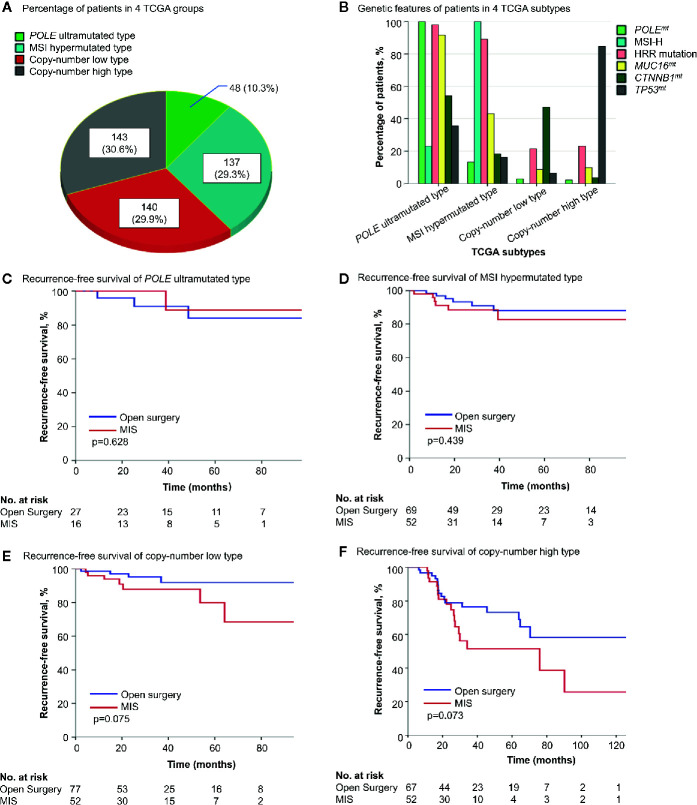
The survival influence of surgical approach in different TCGA molecular subgroups. **(A)** The distribution of 4 TCGA molecular subtypes in the cohort. **(B)** Genetic alterations in each molecular subtype. **(C–F)** Kaplan-Meier survival curves of patients with different molecular features by surgical approach. TCGA, the Cancer Genome Atlas; *POLE^mt^*, *POLE* mutation; MSI-H, microsatellite-instability high; HRR, homologous recombination repair; *MUC16^mt^*, *MUC16* mutation; *CTNNB1^mt^*, *CTNNB1* mutation; *CTNNB1^wt^*, *CTNNB1* wild type; *TP53^mt^*, *TP53* mutation; *TP53^wt^*, *TP53* wild type; MIS, minimally invasive surgery.

To further reveal the impact of surgical approach on different patients, four TCGA molecular subtypes of endometrial cancer (12) were analyzed separately. In patients of *POLE* ultramutated and MSI hypermutated type, where one or more of the four alterations (*POLE^mt^*, MSI-H, HRR mutation and *MUC16^mt^*) presented in most cases, surgical approaches showed no influence on survival. For copy-number low and copy-number high type, still the survival difference between the two surgery groups was not significant enough (*p* = 0.075 and 0.073 for RFS in Kaplan-Meier analysis) (**Figure 2**, **Table S4**). But when further stratification was done based on *CTNNB1* and *TP53* status, MIS was found to be associated with shorter RFS in copy-number low type with *CTNNB1^wt^* and copy-number high type with *TP53^mt^* (*p* = 0.048 and 0.037 in Kaplan-Meier analysis) (**Figure S2**).

### Establishment of Simplified Molecular Classification for Deciding Proper Surgical Approaches

More efforts were made to establish a simplified model for deciding surgical approach based on molecular markers. According to the survival data, in patients with one or more of the four features (*POLE^mt^*, MSI-H, HRR mutation and *MUC16^mt^*), open surgery and MIS group showed similar survival outcomes, regardless of *CTNNB1* and *TP53* status. While for the rest, MIS was associated with shortened RFS (*p* = 0.001 in Kaplan-Meier survival analysis and Cox regression). Further stratification was made among the latter based on *CTNNB1* and *TP53* status, and three subgroups were generated: *CTNNB1* mutant subgroup, *TP53* mutant subgroup, no specific molecular feature subgroup (**Table S5**). Since only in *TP53* mutant subgroup was the prognostic effect of MIS significant (*p* < 0.001 in Kaplan-Meier survival analysis, *p* = 0.001 in Cox regression for RFS, **Table S5**), we combined the other two subgroups together, and got three independent molecular subtypes. Patients with ≥1 of the four features (*POLE^mt^*, MSI-H, HRR mutation and *MUC16^mt^*) were named subtype 1. Among the remaining, patients without specific molecular features were named subtype 2, and those with *TP53* mutations were named subtype 3.

The three molecular subtypes varied greatly in multiple clinicopathological characteristics (**Table 2**). We then compared the baseline features of open surgery and MIS cohort within each molecular subtype (**Table S6**). Since postoperative chemotherapy rate was significantly higher in the MIS group in subtype 3 (*p* = 0.043), PSM was used to adjust for it (matching variable: postoperative chemotherapy). And survival analyses based on the cohorts after PSM (**Table S7**) further confirmed the result that open surgery was associated with longer RFS in this subtype (**Figure S3**).

**Table 2:** Comparison of clinicopathological characteristics among 3 molecular subtypes^a^.

Clinicopathological characteristics	Molecular subtypes			*P* Value^b^
	Subtype 1 (N = 272)	Subtype 2 (N = 110)	Subtype 3 (N = 90)	
Advanced age (≥65y), No./Total No. (%)	115/270 (42.5)	39/110 (35.5)	59/90 (65.6)	<0.001
High BMI (≥28kg/m^2^), No./Total No. (%)	168/259 (64.9)	81/107 (75.7)	60/83 (72.3)	0.095
Advanced stage (stage III-IV), No./Total No. (%)	67/272 (24.6)	25/110 (22.7)	37/90 (41.1)	0.005
High grade (G3), No./Total No. (%)	155/272 (57.0)	29/110 (26.4)	84/90 (93.3)	<0.001
Non-endometrioid histology, No./Total No. (%)	43/272 (15.8)	6/110 (5.5)	61/90 (67.8)	<0.001
Lymph node metastasis, No./Total No. (%)	37/235 (15.7)	10/90 (11.1)	23/74 (31.1)	0.002
Positive peritoneal cytology, No./Total No. (%)	21/205 (10.2)	13/85 (15.3)	18/72 (25.0)	0.009
Deep myometrial invasion, No./Total No. (%)	112/241 (46.5)	39/102 (38.2)	34/77 (44.2)	0.373
Residual disease, No./Total No. (%)	39/226 (17.3)	14/88 (15.9)	11/77 (14.3)	0.824
Postoperative radiotherapy, No./Total No. (%)	115/258 (44.6)	39/109 (35.8)	39/82 (47.6)	0.194
Postoperative chemotherapy, No./Total No. (%)	81/254 (31.9)	28/108 (25.9)	45/79 (57.0)	<0.001

BMI, body mass index. ^a^The total numbers of some characteristics are not in accordance with the heading totals because of missing data. ^b^χ^2^ test.

**Figure 3:**
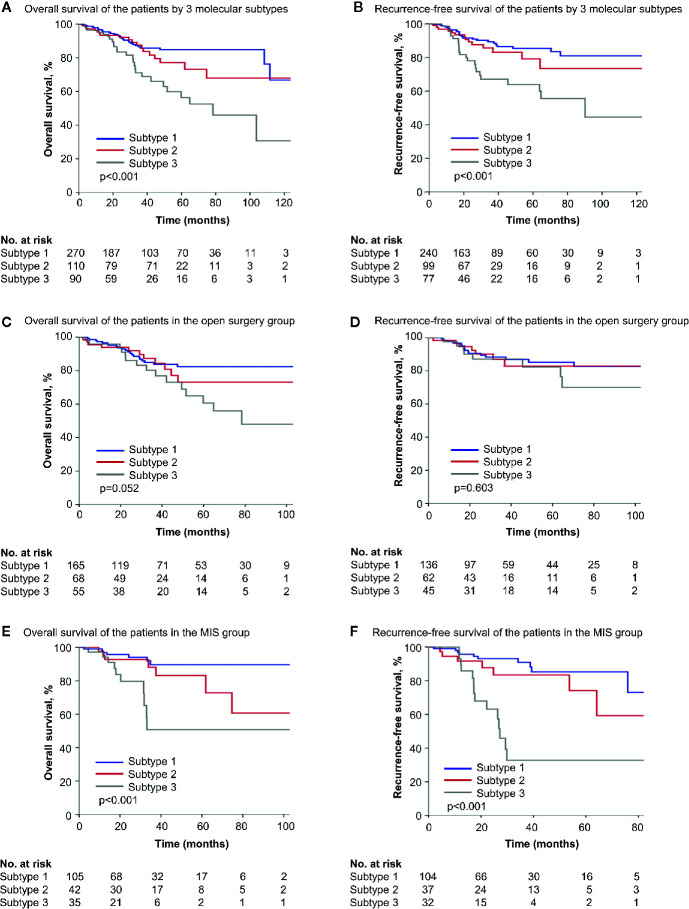
Survival of patients by simplified molecular subtypes. **(A, B)** Kaplan-Meier survival curves of all patients by three molecular subtypes. **(C, D)** Kaplan-Meier survival curves of the open surgery cohort by three molecular subtypes. **(E, F)** Kaplan-Meier survival curves of the MIS cohort by three molecular subtypes. MIS, minimally invasive surgery.

Significant differences in OS and RFS were observed among different subtypes (*p* < 0.001 for OS and RFS in Kaplan-Meier survival analysis, **Figure 3**). And the prognostic difference was influenced by surgical approaches. In the open surgery cohort, OS and RFS were similar (*p* = 0.052 and 0.603, respectively), while in the MIS cohort, three subtypes varied significantly in OS and RFS (*p* < 0.001 for both), with subtype 1 prognostically the best and subtype 3 the worst (**Figure 3**). In univariate Cox regressions, the influence of molecular subtype was significant for both OS and RFS in the MIS cohort, but not in the open surgery cohort (**Table 3**). And in multivariate stepwise regressions, molecular subtype was independently associated with disease recurrence in the MIS cohort (*p* < 0.001) (**Table 4**).

**Table 3:** Univariate Cox regressions for prognostic factors in open surgery and MIS cohorts.

Characteristic	OS		RFS	
	HR (95% CI)	*P* Value	HR (95% CI)	*P* Value
**Open surgery cohort**				
Advanced age (≥65y)	2.105 (1.164-3.808)	0.014	1.364 (0.674-2.760)	0.388
High BMI (≥28 kg/m^2^)	1.262 (0.679-2.346)	0.462	1.910 (0.819-4.456)	0.134
Advanced stage (stage III-IV)	4.467 (2.501-7.980)	<0.001	5.404 (2.620-11.148)	<0.001
High grade (G3)	6.541 (2.589-16.526)	<0.001	5.639 (1.973-16.119)	0.001
Non-endometrioid histology	2.771 (1.568-4.898)	<0.001	2.624 (1.284-5.361)	0.008
Lymph node metastasis	4.327 (2.266-8.262)	<0.001	3.737 (1.636-8.540)	0.002
Positive peritoneal cytology	5.378 (2.702-10.706)	<0.001	5.467 (2.334-12.805)	<0.001
Deep myometrial invasion	3.632 (1.809-7.292)	<0.001	1.881 (0.845-4.187)	0.122
Residual disease	2.565 (1.368-4.807)	0.003	2.858 (1.273-6.421)	0.011
Molecular subtype		0.059		0.608
Subtype 1	reference	–	reference	–
Subtype 2	1.312 (0.634-2.715)	0.465	1.095 (0.449-2.670)	0.842
Subtype 3	2.215 (1.149-4.272)	0.018	1.536 (0.657-3.592)	0.322
**MIS cohort**				
Advanced age (≥65y)	0.901 (0.395-2.056)	0.805	1.247 (0.615-2.529)	0.540
High BMI (≥28 kg/m^2^)	0.858 (0.369-1.994)	0.722	1.057 (0.468-2.388)	0.894
Advanced stage (stage III-IV)	6.452 (2.770-15.026)	<0.001	2.394 (1.135-5.052)	0.022
High grade (G3)	1.920 (0.831-4.437)	0.127	1.349 (0.656-2.774)	0.416
Non-endometrioid histology	5.161 (2.344-11.365)	<0.001	2.224 (1.064-4.650)	0.034
Lymph node metastasis	6.383 (2.437-16.720)	<0.001	3.687 (1.525-8.911)	0.004
Positive peritoneal cytology	3.429 (1.346-8.741)	0.010	2.194 (0.883-5.453)	0.091
Deep myometrial invasion	3.555 (1.459-8.663)	0.005	1.529 (0.736-3.177)	0.256
Residual disease	3.516 (1.245-9.930)	0.018	0.555 (0.117-2.634)	0.459
Molecular subtype		0.001		<0.001
Subtype 1	reference	–	reference	–
Subtype 2	1.638 (0.584-4.594)	0.348	1.868 (0.711-4.911)	0.205
Subtype 3	5.200 (2.069-13.072)	<0.001	6.765 (3.010-15.206)	<0.001

BMI, body mass index; MIS, minimally invasive surgery; OS, overall survival; RFS, recurrence-free survival; HR, hazard ratio; CI, confidence interval.

**Table 4:** Stepwise regressions for independent prognostic factors in open surgery and MIS cohorts.

Characteristic	OS		RFS	
	Adjusted HR (95% CI)	*P* Value	Adjusted HR (95% CI)	*P* Value
**Open surgery cohort**				
Advanced stage (stage III-IV)	3.193 (1.295-7.870)	0.012	3.543 (1.487-8.444)	0.004
High grade (G3)	7.879 (1.826-33.996)	0.006	2.627 (0.865-7.977)	0.088
Positive peritoneal cytology	2.518 (1.048-6.050)	0.039	–	–
**MIS cohort**				
Advanced stage (stage III-IV)	3.927 (1.129-13.662)	0.031	1.988 (0.925-4.272)	0.078
Molecular subtype	–	–		<0.001
Subtype 1	–	–	reference	–
Subtype 2	–	–	1.929 (0.733-5.076)	0.183
Subtype 3	–	–	6.305 (2.782-14.291)	<0.001

MIS, minimally invasive surgery; OS, overall survival; RFS, recurrence-free survival; HR, hazard ratio; CI, confidence interval.

## Discussion

**Figure 4:**
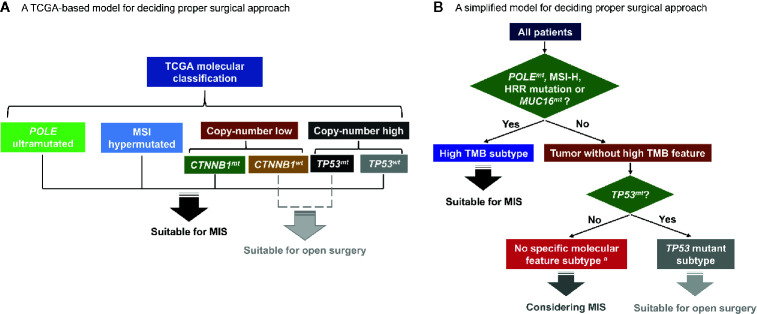
Hypothetical schemas for choosing surgical approaches based on patients’ molecular features. **(A)** A TCGA-based model for deciding proper surgical approach. **(B)** A simplified model for deciding proper surgical approach. TCGA, the Cancer Genome Atlas; MSI, microsatellite-instability; *CTNNB1^mt^*, *CTNNB1* mutation; *CTNNB1^wt^*, *CTNNB1* wild type; *TP53^mt^*, *TP53* mutation; *TP53^wt^*, *TP53* wild type; MIS, minimally invasive surgery; *POLE^mt^*, *POLE* mutation; MSI-H, microsatellite-instability high; HRR, homologous recombination repair; *MUC16^mt^*, *MUC16* mutation; TMB, tumor mutation burden. ^a^ Patients with concurrent *TP53^mt^* and *CTNNB1^mt^* are excluded and should be separately discussed.

Since the TCGA molecular classification of endometrial cancer was proposed (12), some genomic studies have been conducted to establish an integrated molecular risk stratification system (21, 25, 26). And further efforts were made to deliver therapies tailored to certain molecular alterations (27). In this study, we analyzed the influence of surgical approaches on patients in different molecular subgroups. Certain molecular features were proved to influence endometrial cancer patients’ survival after open surgeries or MIS. A TCGA-based model for choosing surgical approaches and a simplified model based on three molecular subtypes were established accordingly (**Figure 4**).

Our previous work has demonstrated that MIS was associated with shorter RFS in microsatellite-stable (MSS) endometrioid endometrial cancer patients compared with the open counterpart, while in other patients, long-term survival in the two surgery groups was similar (14). Surgical manipulations and tumor biological properties, especially elevated mutation load, were thought to account for the difference (14). Recently, compromised DNA damage repair, including mutations in *POLE* exonuclease domain, mismatch repair pathways and HRR pathways, were found to be associated with higher tumor mutation burden (TMB), key clinicopathological features and patients’ prognosis, and could be used for further stratification in endometrial cancer (12, 20, 28–32). In a pan-cancer study by Wang et al. (20), mutations in base-excision repair, mismatch repair, and HRR pathways were demonstrated to be highly correlated with tumor mutation load. And in another recent study, tumors with elevated global mutation load typically showed overall higher mutation rates in multiple DNA damage repair pathways (33). Furthermore, another biomarker, *MUC16^mt^* was proved to be also related with tumor mutation burden and better survival in multiple tumor types, including endometrial cancer (18, 34–36). In our study, the four molecular markers were all included, and their prognostic relevance was examined in detail.

Interestingly, in our study, the above four features (*POLE^mt^*, MSI-H, HRR mutation, and *MUC16^mt^*) were all related with non-inferior survival outcomes after MIS. Based on previous research, weakened tumor cell viability, more neoantigens and stronger antitumor immune response were found to be common features shared by high TMB tumors (20, 28, 29, 37, 38). And peritoneal disseminated tumor cells caused by MIS procedures, which was thought to be one of the sources of disease recurrence (39–41), may be thereby more vulnerable in these circumstances. According to our previous assumption based on tumor microsatellite status (14), which can be generalized to high TMB endometrial cancer, the unique features of such tumors may counterbalance the negative effect of MIS, and results in similar prognosis compared with open surgeries.

In 2015, Stelloo et al. (25) proposed a simplified endometrial cancer classification system using surrogate molecular markers, and identified four prognostic groups including *POLE* mutant, MSI-H, *TP53* mutant, and no specific molecular profile (NSMP) group. In accordance with the TCGA classification, *POLE* mutant and MSI-H group showed relatively better survival outcome and *TP53* mutant group was prognostically inferior (25). Further attempts were made to incorporate genetic, immunohistochemical, and clinicopathological features into the classification system (21). In the risk stratification system described by Stelloo et al. (21), *TP53^mt^* was shown to be one of the unfavorable features, while another molecular marker associated with endometrial cancer recurrence, *CTBBN1^mt^*, was an intermediate risk factor (19, 21).

In our study, *CTNNB1* status also influenced patients’ survival after different surgical approaches in TCGA copy-number low cohort. But in the simplified model, after considering other concurrent molecular alterations, the value of *CTNNB1* in determining suitable surgical approaches was not significant enough. Besides, the survival data of three molecular subtypes in our model was basically consistent with the established models, as mentioned above (21, 25, 26). But a novel finding of our study is that the difference in survival status among three subtypes was greatly influenced by surgical approaches, especially for *TP53* mutant tumors. As shown in our data, patients with *TP53^mt^* (subtype 3) typically showed worse survival than the other two subtypes in the entire cohort. But in the open surgery cohort, similar prognosis was seen, especially as to patients’ RFS, indicating that for *TP53* mutant tumors, the advantage of open surgery was much more prominent. Previous studies demonstrated that *TP53^mt^* in endometrial cancer was associated with more adverse pathological factors, advanced stage and worse survival (21, 25, 26, 42), which was in accordance with our data based on TCGA cohort. And for advanced stage endometrial cancer, the completeness of surgical resection is essential for better prognosis (43, 44). In this regard, for endometrial cancer with *TP53^mt^*, open surgery may be more suitable due to its better intraoperative detection, larger surgical extent and greater number of lymph nodes resected (though not significant enough in our data, see **Table S2**).

In this study, a simplified model based on TCGA data was established, and statistical methods, including PSM and multivariate analyses were conducted for internal validation. Similar to TCGA molecular classification, our model also showed strong associations with clinicopathological parameters, and exhibited good prognostic value. But instead of using multi-omics data, we established the model based on surrogate molecular markers, which enhanced its feasibility in practical use. Besides, the model further helped elucidate the association between tumor molecular features and surgical decisions, providing some novel insights in endometrial cancer treatment.

But some limitations still exist. Firstly, the method of patients’ classification in our model was based on next-generation sequencing (NGS) data, which can hardly be adopted in most medical centers for clinical application, especially in less developed areas. Clinically feasible methods, such as immunohistochemistry, should be studied, and more data are needed to prove the efficacy of surrogate methods. Secondly, in this study, only endometrioid and serous type endometrial cancer were included, further verification of the model in other histological subtypes, like clear cell carcinoma, etc., is essential. Thirdly, as we have pointed out before (14), TCGA as a public database, is limited in inter-case consistency, especially as to surgical manipulations, which may differ among different regions, hospitals and surgeons. Besides, in TCGA cohort, the follow-up time was relatively short, as median OS and RFS were not seen in most subgroups. Further studies based on different cohorts with longer follow-up time are needed as external verification of the results. Finally, though statistical adjustments were conducted, the study was still limited in controlling confounders due to the retrospective nature.

Nowadays, genetic features of endometrial cancer are getting more and more attention from the gynecologic oncology community not only for its significance in identifying patients with higher recurrence risk, but also for selecting patients potentially benefiting from cancer immune and molecular targeted therapies. On one hand, accumulating evidences support the usage of NGS-guided targeted treatment in endometrial cancer patients for its definite survival benefits (27). And immunotherapies, as an example, is getting increasingly wider clinical application, even beyond MSI-H or mismatch repair deficient tumors (45). On the other hand, molecular-based risk stratifications of endometrial cancer are getting closer association with clinical decision-making. In this study, we linked patients’ genetic features to surgical treatment, and, for the first time, established surgical selection models for precise treatment strategy design in endometrial cancer patients harboring certain molecular alterations. Our results, once again, highlighted the vital role of gene testing in clinical cancer treatment and suggested that surgeries, in combination with adjuvant therapy and targeted drugs, could be delivered in an individualized manner. In the future, further refinement of the model is essential, and further attempts of incorporating clinicopathological factors to establish integrated stratification system may be necessary. Besides, as mentioned above, more studies based on prospective cohorts are needed to validate the models in different countries and larger populations.

## Data Availability Statement

Publicly available datasets were analyzed in this study. This data can be found here: https://portal.gdc.cancer.gov/.

## Author Contributions

YD and ZW planned the study. YD, JYW and LZ contributed to methodology, data extraction and formal analysis. YD wrote the original draft. ZW and JLW supervised the whole study, reviewed, and revised the manuscript. ZW and JLW contributed to funding acquisition. All authors contributed to the article and approved the submitted version.

## Funding

This work was supported by the National Natural Science Foundation of China (grant number 81972426, 81874108), Special Projects for Strengthening Basic Research of Peking University (grant number BMU2018JC005), and National Key Technology R&D Program of China (grant number 2019YFC1005200, 2019YFC1005201).

## Conflict of Interest

The authors declare that the research was conducted in the absence of any commercial or financial relationships that could be construed as a potential conflict of interest.
