# Supplementary material for: Tumor Molecular Features Predict Endometrial Cancer Patients’ Survival After Open or Minimally Invasive Surgeries
Source: Front Oncol. 2021 Feb 26;11:634857. doi: 10.3389/fonc.2021.634857 (PMC7952993; doi:10.3389/fonc.2021.634857)
Supplement: Supplementary file 1 [file DataSheet_1.docx]

Supplementary Material

# Supplementary Tables

**Table S1.** Genes in homologous recombination repair related pathways

| Gene names |
| --- |
| *ATM, ATR, BARD1, BLM, BRCA1, BRCA2, BRIP1, CDK12, CHEK1, CHEK2, DNA2, EME1, FANCA, FANCC, FANCD2, FANCE, FANCF, FANCG, FANCI, FANCL, FANCM, MRE11A, MUS81, NBN, PALB2, RAD50, RAD51, RAD51B, RAD51C, RAD51D, RAD52, RAD54L, RAD54B, RBBP8, RMI1, RMI2, RPA1, RPA2, RPA3, SHFM1, SLX1A, SLX4, TOPBP1, TP53BP1, WRN, XRCC2, XRCC3* |

**Table S2.** Lymph node resection in patients receiving different surgical approaches ^a^

| Lymph node regions | Open surgery (N=291) | MIS (N=182) | *P* Value |
| --- | --- | --- | --- |
| Pelvic lymph node resection, No. (%) |  |  | 1.000 ^a^ |
| Yes | 244 (83.8) | 153 (84.1) |  |
| No | 40 (13.7) | 24 (13.2) |  |
| Unknown | 7 (2.4) | 5 (2.7) |  |
| Pelvic lymph nodes resected, mean (SD) | 15.94 (13.71) | 13.92 (10.76) | 0.078 ^b^ |
| Paraaortic lymph node resection, No. (%) |  |  | 0.081 ^c^ |
| Yes | 182 (62.5) | 95 (52.2) |  |
| No | 95 (32.6) | 77 (42.3) |  |
| Unknown | 14 (4.8) | 10 (5.5) |  |
| Paraaortic lymph nodes resected, mean (SD) | 4.86 (6.24) | 4.13 (5.55) | 0.211 ^b^ |

Abbreviations: MIS, minimally invasive surgery. ^a^ Fisher’s exact test. ^b^ Student *t* test. ^c^ χ^2^ test.

**Table S3**. Cox regression analysis of the survival influence of surgical approach on different patient cohorts

| Patient Cohort | OS | | | RFS | | |
| --- | --- | --- | --- | --- | --- | --- |
|  | HR | 95% CI | *P* Value | HR | 95% CI | *P* Value |
| Patients with *POLE^mt^* | 0.027 | 0.000-195.386 | 0.426 | 0.421 | 0.047-3.770 | 0.439 |
| Patients with *POLE^wt^* | 1.089 | 0.668-1.777 | 0.732 | 1.991 | 1.184-3.348 | 0.009 |
| MSI-H patients | 1.117 | 0.405-3.081 | 0.830 | 1.262 | 0.436-3.649 | 0.668 |
| Non MSI-H patients | 0.972 | 0.563-1.680 | 0.920 | 1.988 | 1.130-3.496 | 0.017 |
| Patients with HRR mutation | 0.790 | 0.303-2.057 | 0.629 | 0.940 | 0.368-2.401 | 0.898 |
| Patients with HRR wild type | 1.086 | 0.621-1.898 | 0.773 | 2.427 | 1.332-4.421 | 0.004 |
| Patients with *MUC16^mt^* | 0.778 | 0.199-3.040 | 0.718 | 1.182 | 0.316-4.420 | 0.804 |
| Patients with *MUC16^wt^* | 1.086 | 0.649-1.816 | 0.754 | 2.036 | 1.186-3.492 | 0.010 |
| Patients with *CTNNB1^mt^* | 0.605 | 0.166-2.206 | 0.447 | 0.976 | 0.243-3.917 | 0.972 |
| Patients with *CTNNB1^wt^* | 1.112 | 0.661-1.872 | 0.690 | 1.898 | 1.109-3.250 | 0.019 |
| Patients with *TP53^mt^* | 1.197 | 0.624-2.295 | 0.588 | 2.027 | 1.048-3.919 | 0.036 |
| Patients with *TP53^wt^* | 0.969 | 0.475-1.976 | 0.931 | 1.694 | 0.794-3.613 | 0.172 |

Abbreviations: OS, overall survival; RFS, recurrence-free survival; HR, hazard ratio; CI, confidence interval; *POLE^mt^*, *POLE* mutation; *POLE^wt^*, *POLE* wild type; MSI-H, microsatellite-instability high; HRR, homologous recombination repair; *MUC16^mt^*, *MUC16* mutation; *MUC16^wt^*, *MUC16* wild type; *CTNNB1^mt^*, *CTNNB1* mutation; *CTNNB1^wt^*, *CTNNB1* wild type; *TP53^mt^*, *TP53* mutation; *TP53^wt^*, *TP53* wild type.

**Table S4**. Cox regression analysis of the survival influence of surgical approach based on TCGA molecular classification

| Patient Cohort | OS | | | RFS | | |
| --- | --- | --- | --- | --- | --- | --- |
|  | HR | 95% CI | *P* Value | HR | 95% CI | *P* Value |
| *POLE* ultramutated | 0.026 | 0.000-9.399*10^5^ | 0.681 | 0.576 | 0.060-5.536 | 0.632 |
| MSI hypermutated | 1.037 | 0.376-2.857 | 0.944 | 1.559 | 0.502-4.844 | 0.443 |
| Copy-number low | 0.945 | 0.276-3.232 | 0.928 | 2.906 | 0.849-9.954 | 0.089 |
| Copy-number high | 1.069 | 0.572-1.999 | 0.834 | 1.829 | 0.935-3.577 | 0.078 |

Abbreviations: OS, overall survival; RFS, recurrence-free survival; HR, hazard ratio; CI, confidence interval; MSI, microsatellite-instability.

**Table S5**. Survival data for simplified model of deciding surgical approach based on genetic features

| **Genetic features** | OS by surgical approaches | | | | RFS by surgical approaches | | | |
| --- | --- | --- | --- | --- | --- | --- | --- | --- |
|  | Kaplan-Meier survival analysis ^a^ | Cox regression | | | Kaplan-Meier survival analysis ^a^ | Cox regression | | |
|  | *P* Value | HR | 95% CI | *P* Value | *P* Value | HR | 95% CI | *P* Value |
| ≥**1 of the 4 features ^b^** | 0.339 | 0.674 | 0.299-1.521 | 0.342 | 0.969 | 0.984 | 0.444-2.180 | 0.969 |
| ≥1 of the 4 features and *CTNNB1^mt^* | 0.950 | 1.056 | 0.193-5.783 | 0.950 | 0.981 | 0.971 | 0.087-10.791 | 0.981 |
| ≥1 of the 4 features and *CTNNB1^wt^* | 0.229 | 0.570 | 0.226-1.442 | 0.235 | 0.780 | 0.887 | 0.383-2.056 | 0.780 |
| ≥1 of the 4 features and *TP53^mt^* | 0.388 | 0.576 | 0.162-2.047 | 0.394 | 0.296 | 0.509 | 0.140-1.852 | 0.305 |
| ≥1 of the 4 features and *TP53^wt^* | 0.784 | 0.858 | 0.287-2.567 | 0.784 | 0.311 | 1.751 | 0.584-5.247 | 0.317 |
| **None of the 4 features ^c^** | 0.405 | 1.292 | 0.706-2.364 | 0.406 | 0.001 | 3.006 | 1.551-5.823 | 0.001 |
| *CTNNB1* mutant subgroup | 0.196 | 0.271 | 0.032-2.261 | 0.228 | 0.812 | 0.814 | 0.149-4.450 | 0.812 |
| *TP53* mutant subgroup | 0.126 | 1.854 | 0.831-4.134 | 0.132 | <0.001 | 4.706 | 1.949-11.366 | 0.001 |
| No specific molecular feature subgroup | 0.337 | 1.779 | 0.540-5.856 | 0.343 | 0.128 | 2.919 | 0.689-12.363 | 0.146 |

Abbreviations: OS, overall survival; RFS, recurrence-free survival; HR, hazard ratio; CI, confidence interval; *CTNNB1^mt^*, *CTNNB1* mutation; *CTNNB1^wt^*, *CTNNB1* wild type; *TP53^mt^*, *TP53* mutation; *TP53^wt^*, *TP53* wild type. ^a^ Log rank test. ^b^ 4 features include: *POLE* mutation (*POLE^mt^*), microsatellite-instability high (MSI-H), homologous recombination repair (HRR) pathway mutation, *MUC16* mutation (*MUC16^mt^*). ^c^ 1 case could not be classified into the subgroups because of concurrent *CTNNB1* and *TP53* mutation.

**Table S6.** Clinicopathological characteristics of open surgery and MIS cohorts within each molecular subgroup ^a^

| Clinicopathological characteristics | Molecular subtypes | | | | | | | | |
| --- | --- | --- | --- | --- | --- | --- | --- | --- | --- |
|  | Subtype 1 (N=272) | | | Subtype 2 (N=110) | | | Subtype 3 (N=90) | | |
|  | Open surgery | MIS | *P* Value ^b^ | Open surgery | MIS | *P* Value ^b^ | Open surgery | MIS | *P* Value ^b^ |
| Advanced age (≥65y), No./ Total No. (%) | 70/165 (42.4) | 45/105 (42.9) | 0.944 | 25/68 (36.8) | 14/42 (33.3) | 0.715 | 37/55 (67.3) | 22/35 (62.9) | 0.667 |
| High BMI (≥28kg/m^2^), No./ Total No. (%) | 98/155 (63.2) | 70/104 (67.3) | 0.500 | 52/67 (77.6) | 29/40 (72.5) | 0.551 | 34/49 (69.4) | 26/34 (76.5) | 0.478 |
| Advanced stage (stage III-IV), No./ Total No. (%) | 47/167 (28.1) | 20/105 (19.0) | 0.090 | 15/68 (22.1) | 10/42 (23.8) | 0.831 | 23/55 (41.8) | 14/35 (40.0) | 0.864 |
| High grade (G3), No./ Total No. (%) | 102/167 (61.1) | 53/105 (50.5) | 0.086 | 17/68 (25.0) | 12/42 (28.6) | 0.680 | 52/55 (94.5) | 32/35 (91.4) | 0.674^a^ |
| Non-endometrioid histology, No./ Total No. (%) | 25/167 (15.0) | 18/105 (17.1) | 0.633 | 3/68 (4.4) | 3/42 (7.1) | 0.673^a^ | 39/55 (70.9) | 22/35 (62.9) | 0.426 |
| Lymph node metastasis, No./ Total No. (%) | 27/142 (19.0) | 10/93 (10.8) | 0.089 | 8/57 (14.0) | 2/33 (6.1) | 0.315^a^ | 15/46 (32.6) | 8/28 (28.6) | 0.716 |
| Positive peritoneal cytology, No./ Total No. (%) | 15/119 (12.6) | 6/86 (7.0) | 0.190 | 9/51 (17.6) | 4/34 (11.8) | 0.460 | 9/45 (20.0) | 9/27 (33.3) | 0.206 |
| Deep myometrial invasion, No./ Total No. (%) | 71/140 (50.7) | 41/101 (40.6) | 0.120 | 24/62 (38.7) | 15/40 (37.5) | 0.902 | 19/46 (41.3) | 15/31 (48.4) | 0.539 |
| Residual disease, No./ Total No. (%) | 28/143 (19.6) | 11/83 (13.3) | 0.225 | 11/64 (17.2) | 3/24 (12.5) | 0.750^a^ | 8/49 (16.3) | 3/28 (10.7) | 0.737^a^ |
| Postoperative radiotherapy, No./ Total No. (%) | 68/154 (44.2) | 47/104 (45.2) | 0.869 | 22/68 (32.4) | 17/41 (41.5) | 0.336 | 20/49 (40.8) | 19/33 (57.6) | 0.136 |
| Postoperative chemotherapy, No./ Total No. (%) | 51/150 (34.0) | 30/104 (28.8) | 0.386 | 16/68 (23.5) | 12/40 (30.0) | 0.459 | 23/48 (47.9) | 22/31 (71.0) | 0.043 |

Abbreviations: MIS, minimally invasive surgery; BMI, body mass index. ^a^ The total numbers of some characteristics are not in accordance with the heading totals because of missing data. ^b^ χ^2^ test.

**Table S7.** Clinicopathological characteristics of open surgery and MIS cohorts in molecular subtype 3 after PSM

| Clinicopathological characteristics | Open surgery  (N=31) | MIS  (N=35) | *P* Value |
| --- | --- | --- | --- |
| Advanced age (≥65y), No./ Total No. (%) | 19/31 (61.3) | 22/35 (62.9) | 0.896 |
| High BMI (≥28kg/m2), No./ Total No. (%) | 19/28 (67.9) | 26/34 (76.5) | 0.449 |
| Advanced stage (stage III-IV), No./ Total No. (%) | 15/31 (48.4) | 14/35 (40.0) | 0.493 |
| High grade (G3), No./ Total No. (%) | 28/31 (90.3) | 32/35 (91.4) | 1.000 ^a^ |
| Non-endometrioid histology, No./ Total No. (%) | 25/31 (80.6) | 22/35 (62.9) | 0.111 |
| Lymph node metastasis, No./ Total No. (%) | 9/27 (33.3) | 8/28 (28.6) | 0.702 |
| Positive peritoneal cytology, No./ Total No. (%) | 6/25 (24.0) | 9/27 (33.3) | 0.458 |
| Deep myometrial invasion, No./ Total No. (%) | 12/25 (48.0) | 15/31 (48.4) | 0.977 |
| Residual disease, No./ Total No. (%) | 5/29 (17.2) | 3/28 (10.7) | 0.706 ^a^ |
| Postoperative radiotherapy, No./ Total No. (%) | 16/31 (51.6) | 19/33 (57.6) | 0.632 |
| Postoperative chemotherapy, No./ Total No. (%) | 22/31 (71.0) | 22/31 (71.0) | 1.000 |

Abbreviations: MIS, minimally invasive surgery; BMI, body mass index. ^a^ Fisher’s exact test, all others are by χ^2^ test.

# Supplementary Figures


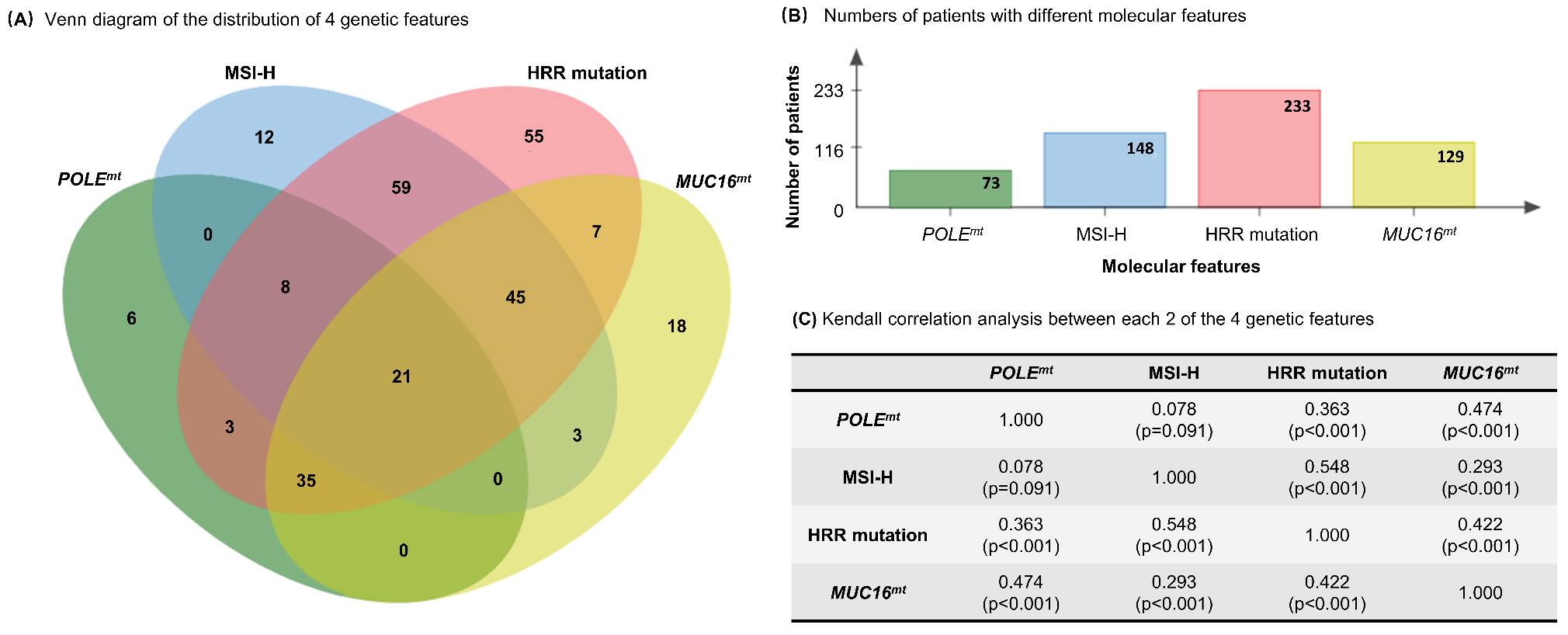


**Figure S1.** Distribution of 4 genetic features among the patients. A. Venn diagram of the distribution of 4 genetic features (*POLE^mt^*, MSI-H, HRR mutation, *MUC16^mt^*). B. Numbers of patients with each molecular feature. C. Kendall correlation analysis between each 2 of the 4 genetic features (numbers in each box indicate the coefficients of correlation, and numbers in the parentheses indicate the p values). Abbreviations: *POLE^mt^*, *POLE* mutation; MSI-H, microsatellite-instability high; HRR, homologous recombination repair; *MUC16^mt^*, *MUC16* mutation.


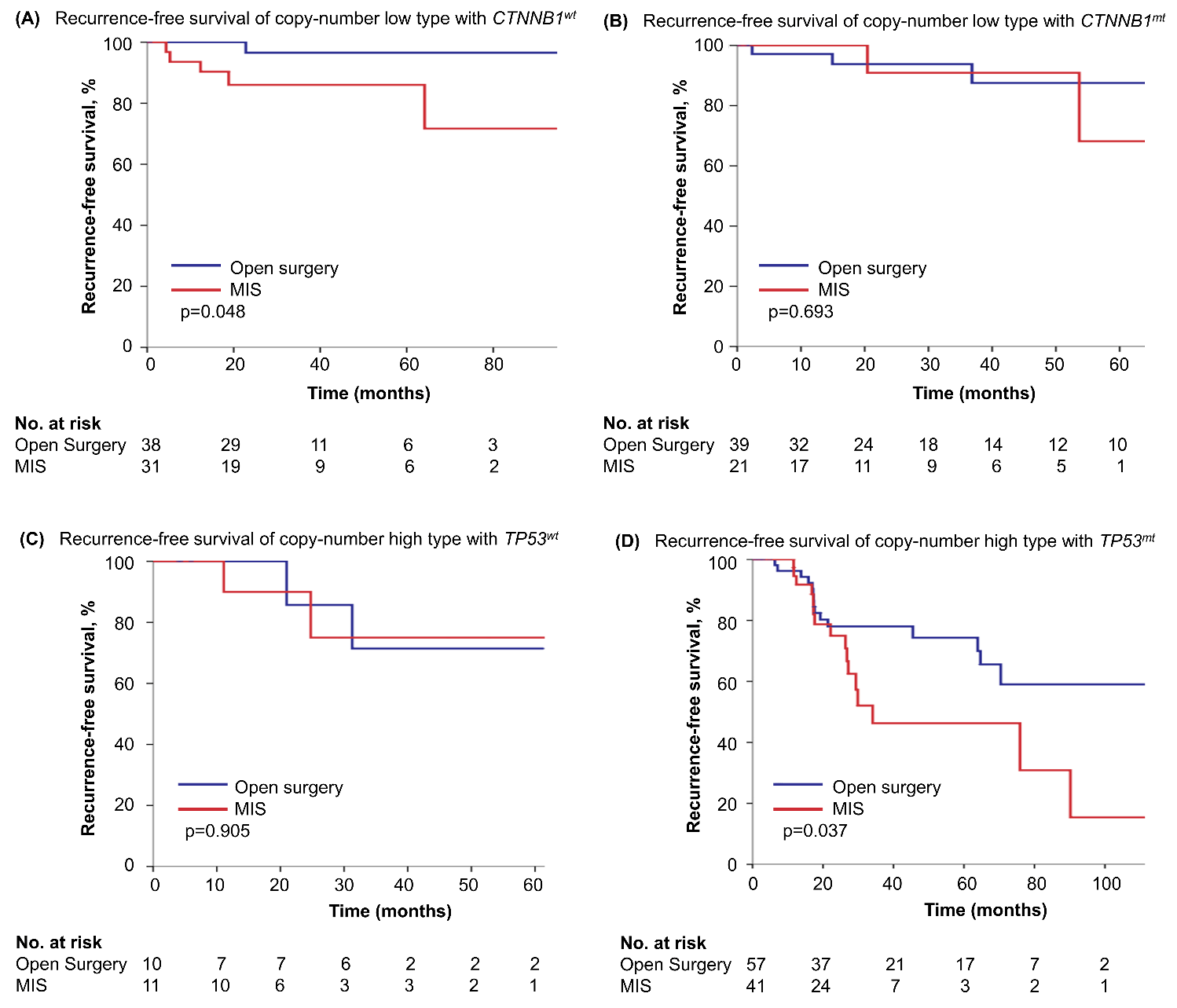


**Figure S2.** The survival influence of surgical approach in TCGA copy-number low and copy-number high subtypes. Abbreviations: TCGA, the Cancer Genome Atlas; *CTNNB1^wt^*, *CTNNB1* wild type; *CTNNB1^mt^*, *CTNNB1* mutation; *TP53^wt^*, *TP53* wild type; *TP53^mt^*, *TP53* mutation; MIS, minimally invasive surgery.


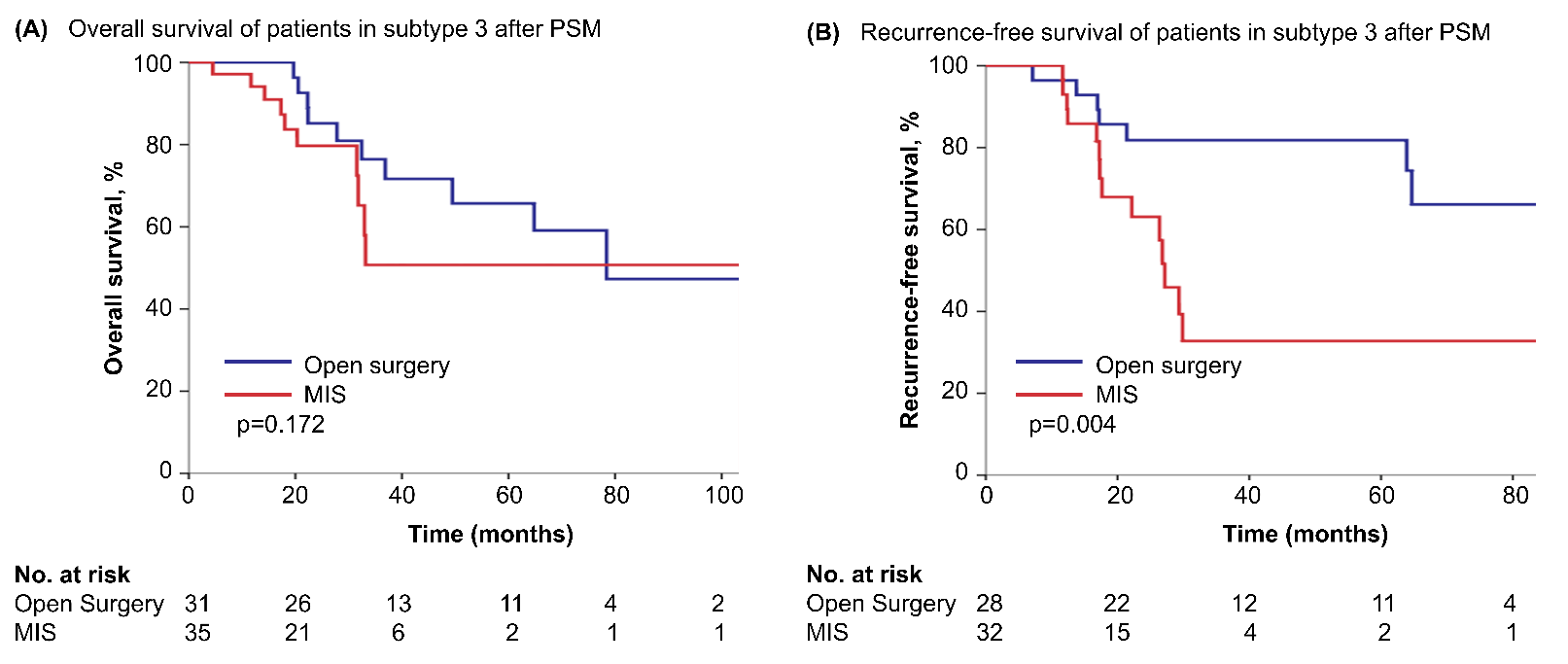


**Figure S3.** The survival influence of surgical approach in subtype 3 after PSM. Abbreviations: PSM, propensity score matching; MIS, minimally invasive surgery.
